# Green Scum

**Published:** 1874-08

**Authors:** 


### GREEN SCUM.

In midsummer, wherever there is stagnant water, a most unsightly and slimy substance, of a green color, is seen floating on the surface, and the general impression is that it is calculated to cause deadly disease. If you put a stick or your finger under it, and raise it up, it hangs in shreds or long lines; on looking at this with a microscope it is found to consist of a mass of living, moving things, called cyclops, having one eye, vibriones, daphnia, etc. These little creatures seem to take no sleep, no rest, but simply eat all the time, growing not an atom, but eventually the green scum, by slow degrees, disappears, the water clears, the busy feasters have all died, leaving nothing to tell that they ever lived, except the little shells which were once their houses.

Many a man lives and dies and leaves not as much a sign of his existence behind as the microscopic vibrione. That is the surface thought; no man lives in vain. He may have had no purpose in life beyond the eating, the gratification of the appetites which characterized an infinitessimal vibrione; but his

Maker has had a purpose in his existence ; he had a work to perform, and his work was done, but having been done, not for the sake of doing good to others, but for the gratification of self, there will be no reward from the Infinite One. While those who take pleasure in helping, in benefiting and blessing others, even the lowest of the race, will live forever in the happy homes above, "Inasmuch as ye did it unto one of the least of these."

These vibriones are really the scavengers of the world ; they eat up what would otherwise bring death to man, and are thus the physical purifiers of the earth.

But the man who lives for noble purposes, a blameless life, and one useful to others, as children of the great Supreme, he is the moral scavenger of the universe, as aiming to eradicate vice and crime wherever they abound, and thus elevate the race to a higher plane, to a more elevated position, a standing place nearer the great Creator of us all.

Reader, are you living like the vibriones which seem to do nothing but feast during their whole existence of a single summer—an existence of self-gratification and indulgence exclusively—or will you resolve "to deny yourself and live to some purpose?" Although the vibrione lives for its own gratification solely, yet its work is overruled for great purposes of good to man. So, however a man may live, his work will be also overruled by the moral Governor of the Universe, to the purification and elevation of society and the race; but having had no other than a selfish aim, he receives no reward—his work is but wood, hay and stubble, the mere scaffolding of the great structure, and, which having subserved its purpose, is destroyed and no more thought of.

On the other hand, the man who lives with the motive of a useful life to others, performs a work which is as gold, and silver, and precious stones, and can never perish, but endures through the ages.

